# The Beneficial Effect of Suramin on Monocrotaline-Induced Pulmonary Hypertension in Rats

**DOI:** 10.1371/journal.pone.0077073

**Published:** 2013-10-15

**Authors:** Mohamed Izikki, Olaf Mercier, Florence Lecerf, Lauriane Lubert Guin, Eric Hoang, Peter Dorfmüller, Frédéric Perros, Marc Humbert, Gerald Simonneau, Philippe Dartevelle, Elie Fadel, Saadia Eddahibi

**Affiliations:** 1 INSERM U999, Le Plessis-Robinson, France; 2 Centre Chirurgical Marie Lannelongue, Le Plessis-Robinson, France; William Harvey Research Institute, Barts and The London School of Medicine and Dentistry, Queen Mary University of London, United Kingdom

## Abstract

**Background:**

Pulmonary hypertension (PH) is a progressive disorder characterized by an increase in pulmonary artery pressure and structural changes in the pulmonary vasculature. Several observations indicate that growth factors play a key role in PH by modulating pulmonary artery smooth muscle cell (PA-SMC) function. In rats, established monocrotaline-induced PH (MCT-PH) can be reversed by blocking platelet-derived growth factor receptors (PDGF-R), epidermal growth factor receptors (EGF-R), or fibroblast growth factor receptors (FGF-R). All these receptors belong to the receptor tyrosine kinase (RTK) family.

**Methods and Results:**

We evaluated whether RTK blockade by the nonspecific growth factor inhibitor, suramin, reversed advanced MCT-PH in rats via its effects on growth-factor signaling pathways. We found that suramin inhibited RTK and ERK1/2 phosphorylation in cultured human PA-SMCs. Suramin inhibited PA-SMC proliferation induced by serum, PDGF, FGF2, or EGF *in* *vitro* and *ex* *vivo*. Treatment with suramin from day 1 to day 21 after monocrotaline injection attenuated PH development, as shown by lower values for pulmonary artery pressure, right ventricular hypertrophy, and distal vessel muscularization on day 21 compared to control rats. Treatment with suramin from day 21 to day 42 after monocrotaline injection reversed established PH, thereby normalizing the pulmonary artery pressure values and vessel structure. Suramin treatment suppressed PA-SMC proliferation and attenuated both the inflammatory response and the deposition of collagen.

**Conclusions:**

RTK blockade by suramin can prevent MCT-PH and reverse established MCT-PH in rats. This study suggests that an anti-RTK strategy that targets multiple RTKs could be useful in the treatment of pulmonary hypertension.

## Introduction

Pre-capillary pulmonary hypertension (PH) is defined by an increase in mean pulmonary artery pressure (Pap) above 25 mmHg in conjunction with a normal pulmonary artery wedge pressure (i.e., less than 15 mmHg). Idiopathic pulmonary arterial hypertension (iPAH) describes chronic PH with no identified cause or associated conditions. It is characterized by an increase in pulmonary vascular resistance that impedes the ejection of blood by the right ventricle (RV) and leads to RV failure and death. The increase in pulmonary vascular resistance is due to pulmonary vascular remodeling, of which an important component is vascular smooth muscle cell (SMC) growth and the consequent increase in the thickness of the wall of the distal pulmonary artery [1]. 

In human patients, as in the experimental models of PH, abnormal proliferation of PA-SMCs is associated with overexpression of certain growth factors, namely platelet-derived growth factor (PDGF), basic fibroblast growth factor-2 (FGF2) and epidermal growth factor (EGF) [2-4]. Binding of these growth factors to their respective receptors leads to PA-SMC proliferation. These receptors belong to the family of transmembrane receptor tyrosine kinases (RTKs). Previous studies [5-7] showed that some drugs, such as Imatinib or Sorafenib, are beneficial but exhibit limited effectiveness in treating human PH, and this is most likely because they only target individual RTKs involved in PH.

In this study, we investigated the effects of suramin, a potent anti-growth factor agent with a broad spectrum of targets, on SMC proliferation in PH. Suramin, an FDA-approved drug, is a symmetrical polysulfonated naphthylamine and a urea derivative. This drug was initially developed as a treatment for trypanosomiasis [7]; however, clinical studies have shown that suramin is an effective anticancer agent [8,9], while it has also been found to have beneficial effects in animal models of muscle, liver and renal interstitial fibrosis [10-12] and in proliferative vitreoretinopathy [13] through its ability to abrogate the phosphorylation of various growth factor receptors.

The purpose of this study was to investigate the effects of suramin on the growth of PA-SMCs that occurs in response to incubation with 10% fetal calf serum (FCS) or growth factors. Using organ culture systems, we also investigated whether suramin could affect the wall thickness of the human pulmonary artery. In addition, we investigated whether suramin is able to attenuate the development of PH or reverse established monocrotaline-induced PH (MCT-PH) in rats, which is characterized by severe PH with no tendency toward spontaneous recovery.

## Methods

### Ethics Statement

This study was approved by the institutional review board and the local ethics committee (Comité de Protection des Personnes, Ile-de-France VII, Le Kremlin-Bicêtre, France). Written, informed consent was given by all the patients prior to their contribution to the study.

Experiments were conducted according to the European Union regulations (Directive 86/609 EEC) for animal experiments and complied with our institution's guidelines for animal care and handling. The animal facility is licensed by the French Ministry of Agriculture (agreement N° B92-019-01). This study was approved by the Committee on the Ethics of Animal Experiments CEEA26 CAPSud. All animal experiments were supervised by Dr. Olaf Mercier (agreement delivered by the French Ministry of Agriculture for animal experiment N° A92–396). All efforts were made to minimize animal suffering.

### Patients

We studied lung specimens obtained from 8 patients during lobectomy or pneumonectomy for localized lung cancer. Preoperative echocardiography was performed to rule out PH, and the lung specimens were collected at a distance from the tumor foci. 

### Isolation, culture, and proliferation tests of human PA-SMCs

PA-SMCs were isolated and cultured as previously described [14]. The cells were subjected to 48 hours of growth arrest in serum-free medium and were then treated with suramin 1 h before incubation with 10% FCS (Eurobio, Courtaboeuf, France). We also tested the effect of suramin on the growth response to exogenous PDGF (10 ng/mL; R&D Systems Europe, Lille, France), EGF (10 ng/mL; R&D Systems Europe, Lille, France), or FGF2 (10 ng/mL, Sigma-Aldrich). For each condition, the cells were incubated for 24 hours and PA-SMC proliferation was then measured by 5-bromo-2-deoxyuridine (BrdU) incorporation and by direct cell counting. 

### Organ culture of human pulmonary arteries

*Ex vivo* organ culture of human pulmonary arteries (hPA) was performed as previously described [15-17]. Briefly, the arteries were obtained from patients, and segments 1 cm in length were prepared for *ex vivo* organ culture. The tissues were then incubated in culture medium that was either unsupplemented or supplemented with 10% FCS, suramin (1000 µg/mL, Sigma-Aldrich, St Louis, MO, USA), or masitinib (10^-5^ M, AB1010, ABscience) for ten days. The segments were fixed in 4% buffered paraformaldehyde and embedded in paraffin before being serially sectioned at 5 μm thickness and prepared for immunostaining and double immunofluorescence staining.

### Receptor tyrosine kinase phosphorylation assay

PA-SMCs cultured in DMEM supplemented with 10% FCS were synchronized for 48 hours. After preincubation with suramin (1000 µg/mL, Sigma-Aldrich) for 1 hour, the cells were stimulated with a combination of PDGF, EGF and FGF2 for 15 minutes at 37°C. The relative levels of tyrosine phosphorylation of the RTKs in the PA-SMCs were determined using the Proteome Profiler™ Human Phospho-RTK Array kit (R&D Systems) in accordance with the manufacturer's protocol. Briefly, cells were lysed in ice-cold lysis buffer and 150 µg of total protein was used for the assay. Densitometric quantification of the immunoblot dots was performed using semi-automated image analysis (ImageJ 1.41).

### Western blotting assay

PA-SMCs were lysed on ice with a buffer containing 20 mM Tris (pH 7.5), 150 mM NaCl, 1 mM EDTA, 1 mM EGTA, 1% Triton X-100, 2.5 mM sodium pyrophosphate, 1 mM β-glycerolphosphate, 1 mM Na_3_VO_4_, and 1 µg/mL leupeptin freshly supplemented with 1 mM phenylmethylsulfonyl fluoride (PMSF). The protein concentration was determined using the Bradford protein assay (Bio-Rad Laboratories, Richmond, CA, USA). Samples containing 10 µg proteins were fractionated by 10% sodium dodecyl sulfate polyacrylamide gel electrophoresis and transferred to nitrocellulose membranes. ERK1/2 was then detected using a rabbit anti-ERK1/2 polyclonal antibody (Ozyme, Saint-Quentin Yvelines, France) diluted 1:300 in 1% milk. The secondary antibody was a polyclonal antibody and was used at a dilution of 1:10000 (Calbiochem, Fontenay-sous-Bois, France). Immunoreactive bands were visualized using chemiluminescence (ECL) (GE Healthcare) on a Bio-Rad Fluoro-S-Max Chemidoc system. For each sample, total ERK levels were also estimated using the rabbit polyclonal ERK antibody (1:2000). A polyclonal antibody against β-actin (diluted 1:3000; Sigma Aldrich) served as the internal control. Densitometric quantification of the immunoblot bands was performed using Bio-Rad Quantity One software.

### Flow cytometry assessment of apoptosis

Apoptosis was detected using the Annexin V-fluorescein isothiocyanate (FITC) Apoptosis Detection Kit I (BD Biosciences, Le-Pont-de-Claix, France). PA-SMCs were treated with suramin (1000 µg/mL). After 24 h, the culture medium containing the detached cells was collected. The plates were rinsed with phosphate buffered saline (PBS), and the cells were detached using 0.05% trypsin/EDTA and combined with their medium and floating cells. The cells were washed twice in cold PBS and resuspended at a density of 10^6^ cells/mL in the binding buffer provided. Each sample was incubated with 5 µL of each of the provided Annexin V-FITC and propidium iodide (PI) solutions for 15 min in the dark. The sample volumes were then increased to 500 µL, and the samples were run using CyAn (Dako-Cytomation, Trappes, France). 

### Treatment of animals with suramin

For all experiments, we used adult male Wistar rats (200-225 g) from Charles River (Les Oncins, France). Animal procedures and care followed institutional guidelines that complied with national and international regulations. Pulmonary hypertension was induced by a single subcutaneous injection of monocrotaline (60 mg/Kg). Assessment of pulmonary hypertension was performed as previously described [4]. Briefly, a polyvinyl catheter was introduced into the right jugular vein, then pushed through the RV into the pulmonary artery. A polyethylene catheter was inserted into the right carotid artery. PAP and systemic artery pressure were measured, the thorax was opened, and the left lung was immediately removed and frozen in liquid nitrogen. The heart was dissected and weighed for calculation of the RV hypertrophy index (RV/[LV + S]). The right lung was fixed in the distended state with formalin buffer. After routine processing and paraffin embedding, multiple sections from each lobe were stained with H&E. In each rat, 60 intra-acinar arteries were examined and categorized as nonmuscular (NM), partially muscular (PM), fully muscular (FM), or obliterated (FM+). 

To assess the potential preventive and curative effects of suramin, rats were randomly divided into four groups after MCT injection. In the preventive strategy, the treatment was started on the first day, and one group received 10 mg/kg suramin intravenously twice weekly for 3 weeks [18,19], while a second group received only the vehicle at the same time points. To assess the potential curative effects of suramin, rats were given MCT and were left untreated for 21 days before being randomly divided into two groups that were subsequently treated with either suramin or vehicle from day 21 to day 42 inclusive. The effect of suramin on survival was evaluated from the day 21 of MCT injection to day 42 corresponding to the treatment period.

### Immunostaining and double immunofluorescence staining

Tissue sections were deparaffinized in xylene and then treated with a graded series of alcohol washes before being rehydrated in PBS (pH 7.5) and incubated with target retrieval solution (Dako, Glostrup, Denmark) in a water bath at 90°C for 20 minutes.

Endogenous peroxidase activity was blocked with H_2_O_2_ in PBS (3% v/v) for 5 minutes. The slides were washed with PBS and incubated for 30 minutes in a protein-blocking solution. The slides were then incubated for 30 minutes with anti-PCNA mouse monoclonal antibody (PC-10, Dako) or anti-CD68 antibody (HyCult Biotechnology, Uden, The Netherlands) at a dilution of 1:200. The slides were processed using the alkaline phosphatase LSAB+ system horseradish peroxidase detection kit (Dako). Brown staining was generated using a diaminobenzidine substrate, and the nuclei were counterstained with hematoxylin. To assess matrix accumulation, the sections were stained with collagen-specific Masson trichrome dye. 

For double immunofluorescence staining, the human pulmonary artery sections were incubated with secondary antibodies labeled with either Alexa Fluor 488 or Alexa Fluor 594 (Invitrogen, Carlsbad, CA) for 1 h at room temperature. After being washed with PBS, the tissue sections were counterstained with 4,6-diamidino-2-phenylindole (Vector Laboratories). Images were captured using a charge-coupled device Iris camera (CCD-Iris, Sony, Japan) in conjunction with a light microscope (Leitz Laborlux, Wetzlar, Germany).

### Statistical analysis

All results were reported as the mean plus or minus the SEM. 

For studies performed on PA-SMCs, the nonparametric Mann-Whitney test was used for comparisons between groups.

For animal studies, comparisons of data obtained at various times after MCT injection or from the various treatment groups were performed using the nonparametric Kruskal-Wallis test followed, where appropriate, by Dunn’s test. Kaplan-Meier methods were used to obtain survival curves, and a two-sided log-rank test was used to compare strata. To compare the degree of pulmonary vessel muscularization between groups, we used a nonparametric Mann-Whitney or Kruskal- Wallis test after ordinal classification of the vessels as nonmuscular (NM), partially muscular (PM), fully muscular (FM), or obliterated (FM+). 

## Results

### The effects of suramin on PA-SMC proliferation and apoptosis

**Figure 1:**
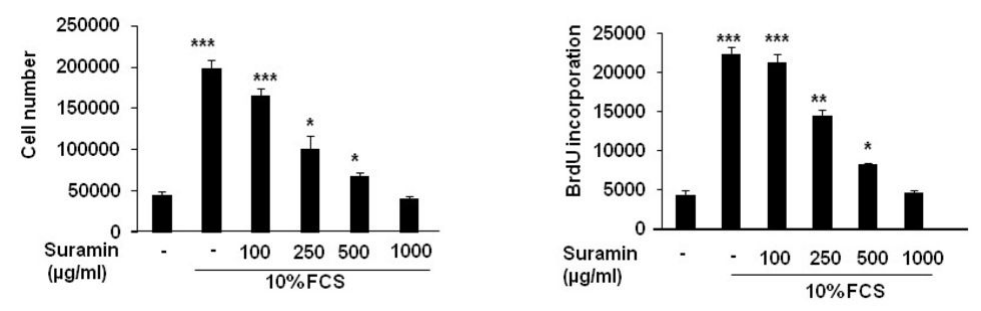
BrdU incorporation and changes in cell number in human pulmonary-artery smooth muscle cells in response to 10% fetal calf serum in the presence or absence of a range of concentrations of suramin (100 to 1000 µg/mL). n=4; *P<0.05; **P<0.001; ***P<0.0001.

We used BrdU incorporation assays and direct cell counting to investigate the ability of suramin to inhibit cell proliferation. The cultures of human PA-SMCs in medium supplemented with 10% FCS were treated with suramin at final concentrations of 100, 250, 500 and 1000 µg/mL for 24 hours. Both the degree of BrdU incorporation observed and the cell counts obtained indicated an inhibition of PA-SMC growth by suramin (Figure 1). The 1000 µg/mL concentration was used in all subsequent experiments. 

**Figure 2:**
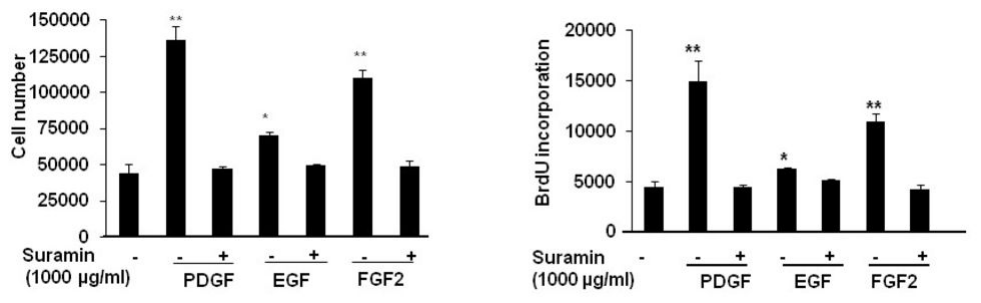
The effect of suramin on growth factor-induced pulmonary-artery smooth muscle cell proliferation. BrdU incorporation and cell count data are shown for quiescent cells incubated with PDGF, FGF2, or EGF (10 ng/mL). Suramin (1000 µg/mL) was added 1 hour before growth factor addition. n=4; *P<0.05; **P<0.001; ***P<0.0001.

As shown in Figure 2, addition of PDGF, FGF2 or EGF (10 ng/mL) to the medium increased both BrdU incorporation and the number of cells, but these effects were completely abolished when the cells were pretreated with suramin. The doses of suramin applied had no toxic effect; indeed, cell viability and apoptosis were similar in the cells treated with suramin and those incubated with the vehicle alone (Live cells: 81.0 +/- 3.7 vs 82.7 +/- 0.3%; Total apoptosis: 14.7 +/- 0.9 vs 13.3 +/- 2.3%; Early apoptosis: 9.0 +/- 1.1 vs 10.3 +/- 2.3%; Late apoptosis: 5.7+/- 0.7 vs 3.0 +/- 2.1%; Necrosis: 4.3 +/- 2.9 vs 4.0 +/- 2.5%, respectively PA-SMC vs PA-SMCs + suramine (1000 µg/mL)).

### Suramin inhibits growth-factor RTK activation in PA-SMCs

**Figure 3:**
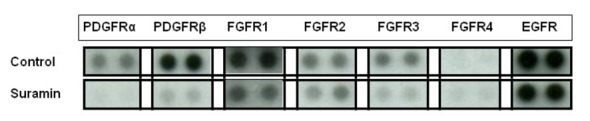
Differential expression of phosphorylated growth factor receptor tyrosine kinases (RTKs) in PA-SMCs. Arrays were incubated with 150 µg of lysate prepared from untreated cells or cells treated with suramin (1000 µg/mL, 1 h) and incubated for 15 minutes with 10 ng/mL of PDGF, FGF2, or EGF. RTKs were analyzed with anti-phospho-RTK, while immunoglobulin (IgG) and "reference spots" were used as the negative and positive controls, respectively. n=4.

Using phospho-receptor tyrosine kinase arrays, we assessed the effect of suramin on the pattern of growth-factor-stimulated RTK phosphorylation in PA-SMCs in response to a combination of PDGF, FGF2 and EGF (Figure 3). As shown in the dot blot, pretreatment of cells with suramin induced a marked decrease in the phosphorylation of PDGF-Rα, PDGF-Rβ, FGF-R1 and EGFR compared to that of cells incubated in the presence of the growth factors alone. The inhibition of receptors phosphorylation was greatest for the PDGF receptors α and β (PDGF-Rα, 41 +/- 3.51%; PDGF-Rβ, 52 +/- 2.70%, respectively), than for FGFR1 (23 +/- 2.31%) or EGFR (10 +/- 1.20%).

### The effect of suramin on growth factor-induced ERK1/2 phosphorylation and cell proliferation

**Figure 4:**
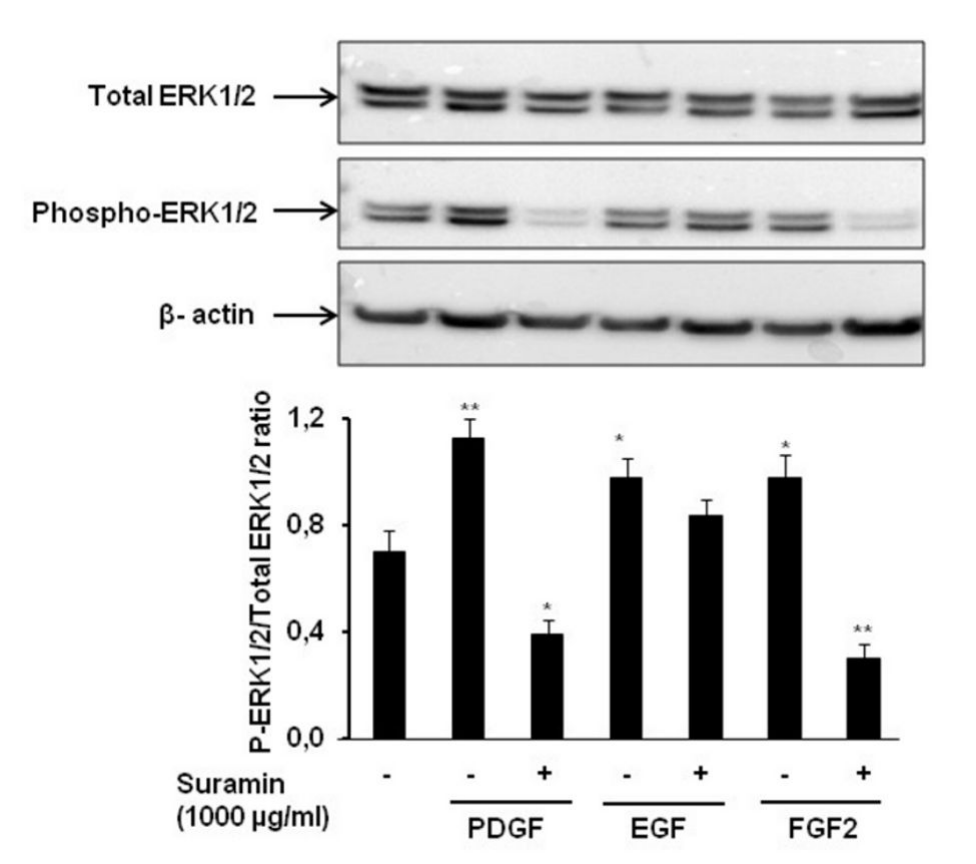
The effect of suramin on PDGF, FGF2, and EGF-induced ERK1/2 phosphorylation in PA-SMCs. Western blotting was used to assess ERK1/2 phosphorylation in PA-SMCs incubated with PDGF, FGF2, or EGF (10 ng/mL, 10 min) with or without suramin pretreatment (1000 µg/mL, 1 h). The results shown are typical findings from five independent experiments. The level of phosphorylated ERK1/2 was normalized against β-actin. n=4. *P<0.05 versus control; **P<0.001 versus control. The phospho/total ratio was expressed as the mean±SEM from five PA-SMC samples.

As ERK phosphorylation is an indication of cell proliferation, we investigated the effect of suramin (1000 µg/mL) on ERK1/2 status in PA-SMCs exposed to PDGF, FGF2 and EGF. As illustrated by Figure 4, PDGF, FGF2, and EGF treatment resulted in rapid ERK1/2 activation. Suramin significantly suppressed the phosphorylated ERK1/2 which was more marked when cells were stimulated with PDGF and FGF2. However, total ERK1/2 levels were not affected by treatment of PA-SMCs with suramin (1000 μg/mL).

### Vascular remodeling in an organ culture model of the human pulmonary artery

**Figure 5:**
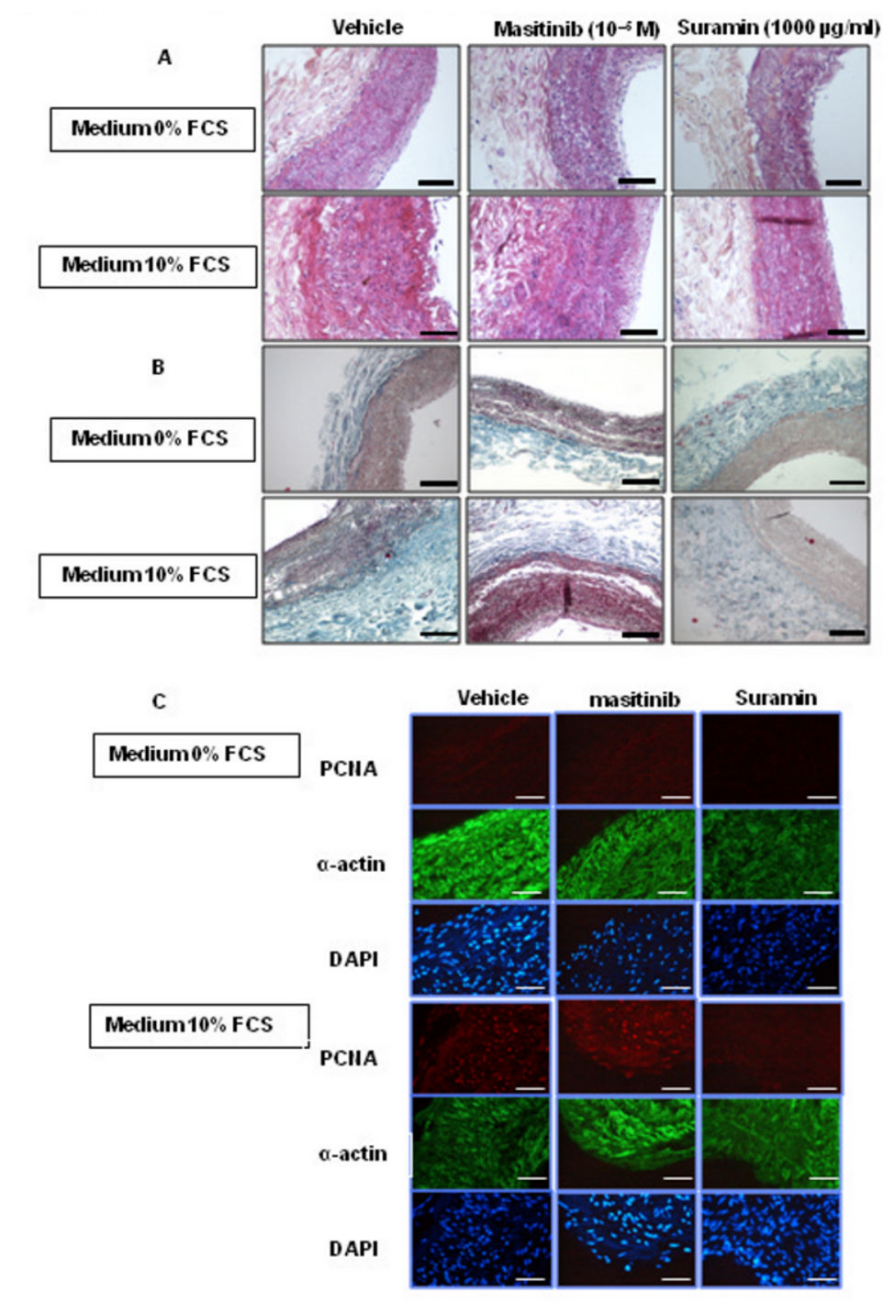
The structure and wall thickness of human pulmonary artery segments after 10 days of incubation in culture medium. The culture medium was either unsupplemented or supplemented with 10% FCS, suramin (1000 µg/mL) or masitinib (10^-5^ M). (**A**) Hematoxylin-phloxine-saffron stain Scale bars: 25 µm (**B**) Masson trichrome stain Scale bars: 25 µm. (**C**) Double immunofluorescence staining performed with an anti-proliferating cell nuclear antigen (PCNA) antibody (red signal) and an anti-α-smooth muscle actin (α-SMA) antibody (green signal). DAPI nuclear staining is also shown (blue signal). Scale bars: 50 µm.

Human pulmonary arteries were incubated in medium with or without 10% FCS in the presence of either suramin (1000 µg/mL) or masitinib (10^-5^ M), and vessel wall thickening was measured at the end of the incubation period. As shown in Figure 5, incubation of PA tissue in the 10% FCS medium induced an increase in wall thickness (a 2.5-fold increase compared with PA tissue incubated in serum-free medium). Inclusion of suramin in the medium dramatically inhibited the arterial remodeling (Figure 5A). In contrast, masitinib, a PDGF receptor inhibitor, induced a slight reduction in the vascular wall thickness, but this was not found to be statistically significant. 

The effect of suramin was due to an inhibition of both extracellular matrix accumulation (Figure 5B) and cell growth assessed by PCNA labeling. Indeed, we did not observe PCNA-positive cells in the walls of the pulmonary artery explants that were incubated in the presence of Suramin (Figure 5C).

### Prevention of MCT-induced PH by suramin

**Figure 6:**
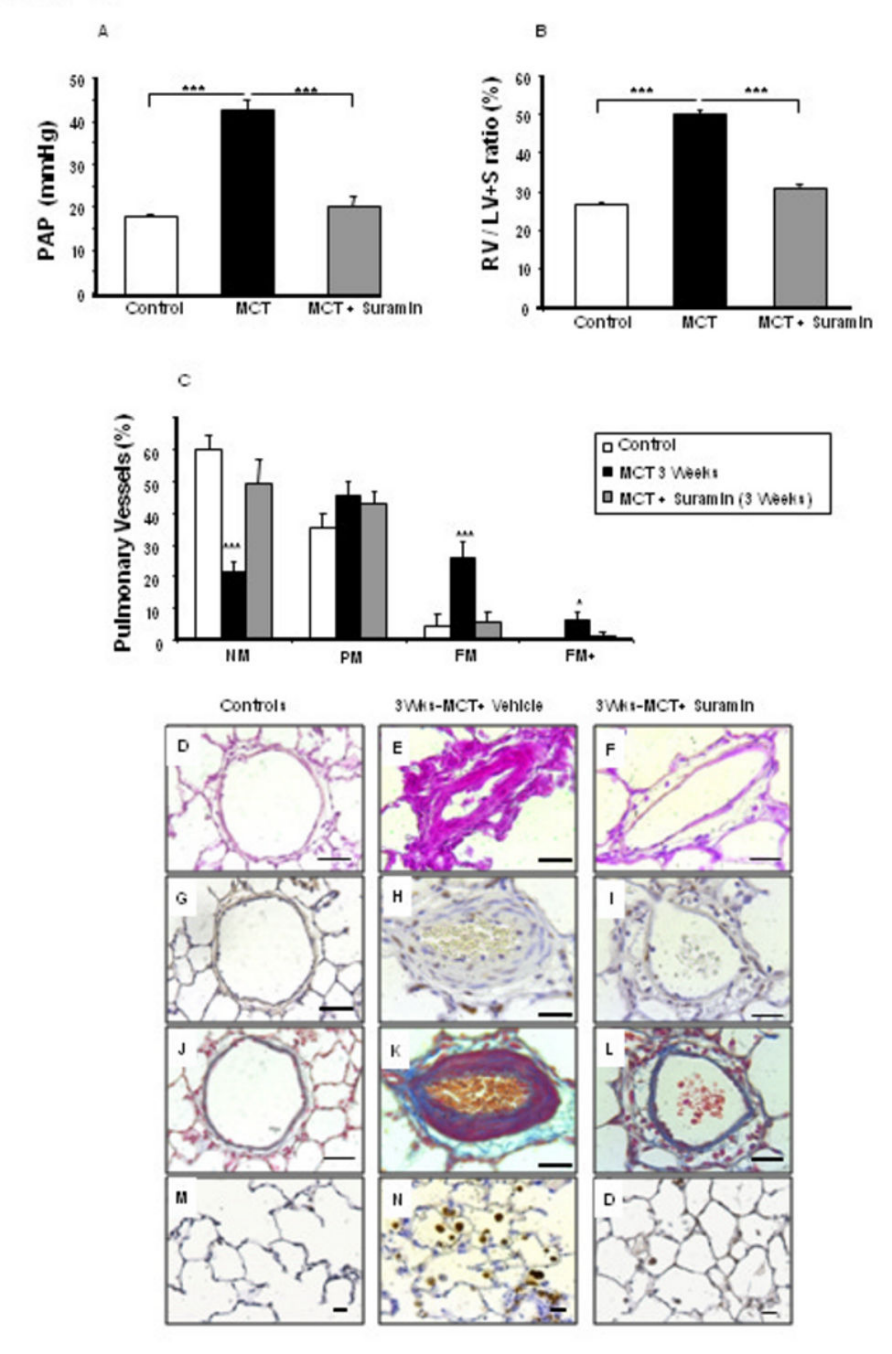
The preventive effect of suramin on the development of monocrotaline-induced pulmonary hypertension. Compared with vehicle administered alone, suramin treatment significantly prevented the development of pulmonary hypertension and right ventricular hypertrophy. **A**) Pulmonary arterial pressure measurements. **B**) Right ventricular hypertrophy as assessed by the RV/(LV+S) weight ratio. **C**) Percentages of nonmuscular (NM), partially muscular (PM), fully muscular (FM), and completely obliterated (FM+) intra-acinar vessels. All values are the mean±SEM from at least 5 animals per group. **D**-**O**) Histochemical and immunohistochemical analysis of rat lung tissue at 21 days after monocrotaline injection. Medial hypertrophy was associated with an increased number of proliferating vascular cells shown by immunohistochemistry for PCNA; PCNA-positive cells have dark nuclei. Suramin prevented the development of medial hypertrophy and the deposition of collagen around the pulmonary arteries. **D**-**F**) Hematoxylin-phloxine-saffron staining. **G**-**I**) Proliferation of pulmonary artery smooth muscle cells**. J**-**L**) Collagen deposition. **M**-**O**) Macrophages in the lung tissue. All values are the mean±SEM from at least 5 animals in each group. *P<0.05; **P<0.001; ***P<0.0001. The statistical analyses compared MCT-injected rats (no suramin treatment) vs. rats injected with saline instead of MCT or rats treated with suramin. Scale bar=25 µm in all sections.

When examined at 21 days after MCT administration, the animals had developed PH (illustrated by a marked increase in pulmonary artery pressure (PAP)), RV hypertrophy (assessed by the ratio of RV weight over LV and interventricular septum (S) weight (RV/[LV + S])) and distal pulmonary artery muscularization, and these changes were not seen in control rats injected with saline instead of MCT (Figure 6A, 6B and 6C). However, the severity of these changes was reduced in animals that were treated chronically with suramin from day 1 to day 21 after MCT injection, and this occurred in the absence of significant effects on body weight, systemic artery pressure, or heart rate (Table 1).

**Table 1:** Body weight, systemic arterial pressure, and heart rate in rats treated with vehicle or suramin (10 mg kg^-1^ day^-1^) between days 21 and 42 after monocrotaline administration.

		**Body weight**	**SAP**	**HR**
		**(g)**	**(mm Hg)**	**(beats/min)**
**3 weeks**	**MCT**	270 ± 10	120 ± 9	392 ± 29
**3 weeks**	**Suramin**	260 ± 4	119 ± 2	395 ± 18
**6 weeks**	**MCT**	328 ± 18	122 ± 7	363 ± 30
**6 weeks**	**Suramin**	343 ± 13	127 ± 6	408 ± 24

All values are the mean±SEM.

MCT-induced PH in rats is related to PA-SMC proliferation, collagen accumulation, and inflammation. Therefore, we evaluated the effect of suramin treatment on these three processes. Immunohistochemical analysis of proliferating cell nuclear antigen (PCNA) indicated that suramin markedly reduced SMC proliferation within the arterial wall (Figure 6 G-I). Moreover, suramin treatment was found to reduce both the accumulation of collagen fibers (which are stained blue by Masson trichrome stain) (Figure 6 J-L) and the infiltration of macrophages (as assessed by immunohistochemistry using the macrophage-specific marker CD68) (Figure 6 M-O) when treated animals were compared with vehicle-injected control rats. 

### Reversal of MCT-Induced PH by suramin

Survival was examined in two groups of rats treated from day 21 to day 42 after MCT. On day 42, 10 of 15 rats (67%) treated with suramin (10 mg/kg) were alive compared with 12 of 25 rats (48%) treated with vehicle (*P*< 0.05).

**Figure 7:**
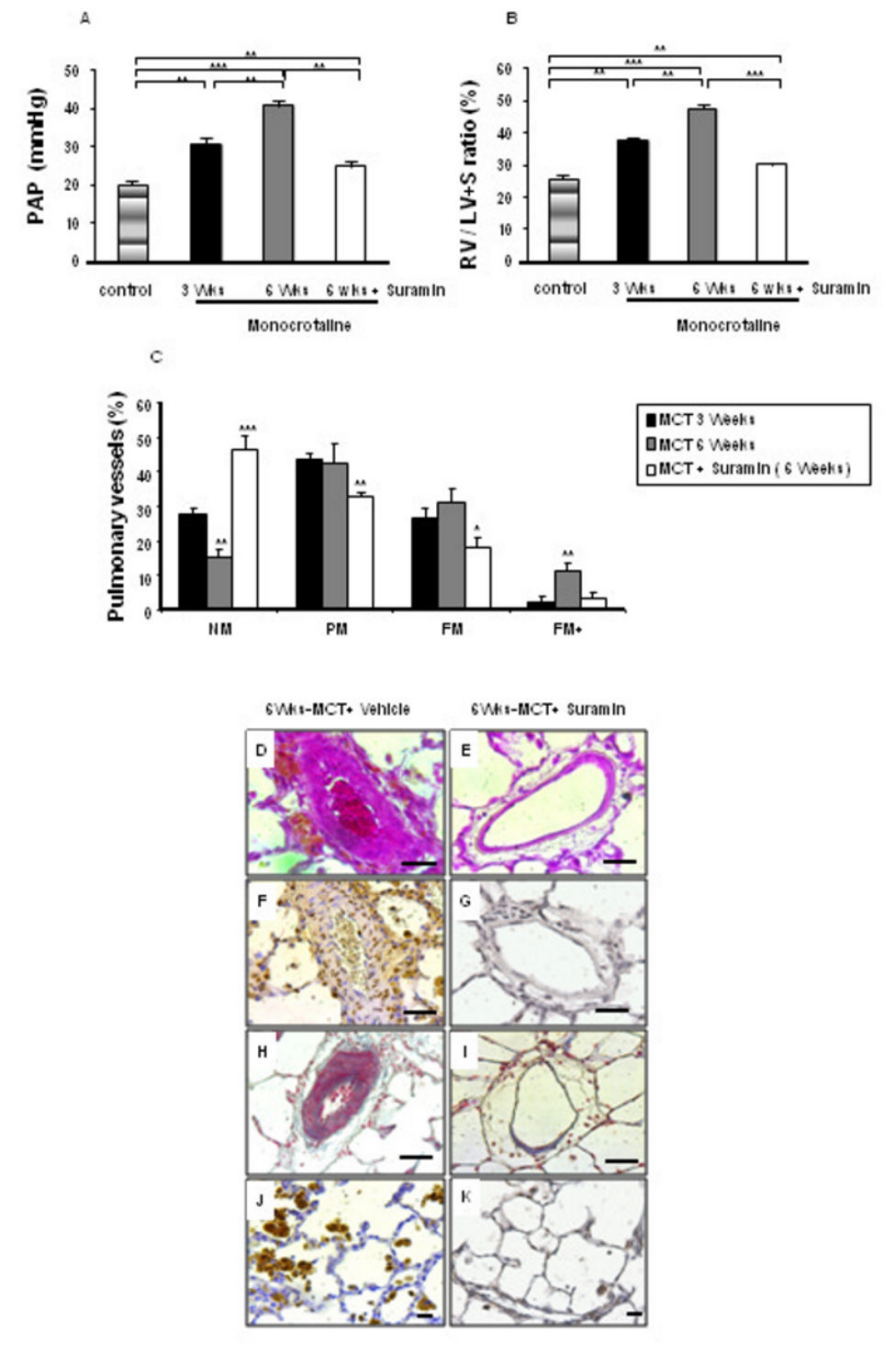
The curative effect of suramin on established monocrotaline-induced pulmonary hypertension. Suramin or vehicle was administered to Wistar rats from day 21 through day 42 following monocrotaline injection. **A** and **B**) Pulmonary artery pressures and right ventricular hypertrophy as assessed by the RV/(LV+S) weight ratio. Compared to vehicle, suramin significantly reversed the pulmonary arterial hypertension and right ventricular hypertrophy. **C**) The percentages of nonmuscular (NM), partially muscular (PM), fully muscular (FM), and completely obliterated (FM+) intra-acinar vessels. All values are the mean±SEM from at least 6 animals. Compared to vehicle administered alone, suramin significantly decreased distal artery muscularization. **D**-**K**) Histochemical and immunohistochemical analysis of rat lung tissue at 42 days after monocrotaline injection and with suramin or vehicle administered from day 21 to day 42. **D** and **E**) Hematoxylin-phloxine-saffron stain. **F** and **G**) Proliferation of pulmonary-artery smooth muscle cells. **H** and **I**) Collagen deposition. **J** and **K**) Macrophages in the lung tissue. Suramin markedly reduced PA-SMC proliferation assayed by PCNA staining (**G**), collagen deposition (**I**), and macrophage infiltration (**K**) around the pulmonary arteries. *P<0.05; **P<0.001; ***P<0.0001. The statistical analyses compared MCT-injected rats (no suramin treatment) vs. rats injected with saline instead of MCT or rats treated with suramin. Scale bar=25 µm in all sections.

Rats given suramin after the development of MCT-PH, from day 21 to day 42, had significantly lower Pap (Figure 7A) and RV/(LV+S) (Figure 7B) values and significantly reduced distal pulmonary artery muscularization (Figure 7C) compared to control animals at day 42. Indeed, in MCT-treated rats given vehicle instead of suramin, the RV/(LV+S) value increased from 37.5±1 to 48±1.5 (*P*<0.05) between days 21 and 42. Systemic artery pressure and heart rate in suramin-treated rats on day 42 were not significantly different from those in control rats given saline instead of MCT (Table 1).

Clearly the effect of suramin was related to a decrease of PA-SMC proliferation (Figure 7F-G), collagen fiber accumulation (Figure 7H-I), and macrophage infiltration (Figure 7J-K) in treated rats compared with those injected with the vehicle alone. 

## Discussion

Our results showed that suramin exerted a very potent inhibitory effect on PA-SMC proliferation induced either by serum or by activation of RTK receptors by PDGF, FGF2 or EGF. The mechanism by which suramin inhibited vascular hyperplasia involves the blockade of RTK activation and ERK1/2 signaling. This effect was also observed in our organ culture system in which treatment of human pulmonary arteries *ex vivo* with suramin, in the presence of serum, was found to inhibit vascular remodeling significantly when compared with the effect of masitinib treatment. The beneficial effect of suramin on pulmonary vascular remodeling was also evident in the results of our in vivo studies performed on rats with MCT-induced PH. In this experimental model, we demonstrated that suramin prevented and reversed PH induced by MCT, improved survival, and animals treated continuously with suramin have a lower PAP, reduced ventricular hypertrophy, and reduced vascular remodeling in comparison to the control animals exposed to the same protocol but treated with vehicle instead of suramin.

The current therapeutic approaches for the treatment of PH mainly provide symptomatic relief in addition to some improvement of prognosis. Recently, several growth factors, including PDGF, FGF2, EGF and others, and growth factor receptor tyrosine kinases (RTK) have been implicated in the abnormal proliferation and migration of PA-SMCs [2-4]. Tests carried out in human patients and in experimental models of PH on drugs that inhibit individual factors showed that they have a beneficial but limited effect. An example of this is provided by studies of imatinib [5,6], which is a selective antagonist of PDGF. Because pulmonary hypertension is associated with the overexpression or activation of several RTK receptors, including those for PDGF, FGF2 and EGF, our study aimed to target the majority of these RTK receptors through Suramin treatment. Suramin is a polyanionic compound originally designed as a drug for treating trypanosomiasis and onchocerciasis [7]. It is well known that suramin is a unique anti-growth factor agent with a broad spectrum of targets. PDGF, EGF, TGFβ, FGF2, IGF are all known to be inhibited by suramin [20]. These unique pharmacological properties may be the reason why suramin has shown an antiproliferative effect against advanced cancers of the prostate, kidney, adrenal gland, and ovary, as well as lymphoma in clinical trials [8,9]. Suramin has also exhibited efficacy as a treatment in animal models of fibrosis [10-12] and proliferative vitreoretinopathy [13].

In this study, we examined the potential of suramin to treat PH by utilizing three different experimental settings; (I) cultured human PA-SMCs, (II) human pulmonary artery organ culture and (III) rats with PH induced experimentally by MCT injection. In cultured cells, suramin induced a dose-dependent inhibition of serum-stimulated PA-SMC proliferation. A suramin concentration of 1000 µg/mL was sufficient for complete neutralization of the proliferation. Other studies on cancerous cells [21], CHO cells [22], and chick chorioallantoic membrane [23] showed that suramin at a dose greater than 500 µg/mL completely inhibited cell proliferation, and this is in agreement with our results in PA-SMCs. The effect of suramin on PA-SMCs was not related to a toxic effect; indeed, exposure of the cells to suramin (1000 µg/mL) for 24 hours had neither apoptotic nor necrotic effects (data not shown). This finding is in agreement with those reported by Zanghi et al. [24] who demonstrated that cell viability was unaffected in suramin-protected CHO cells. 

Growth factors mediate diverse biological responses by binding to RTKs that transmit signals regulating cell proliferation, differentiation, cell migration and survival. Growth factor binding activates RTKs by inducing receptor dimerization. The first response to the binding of growth factors is autophosphorylation of RTKs and the recruitment and activation of a host of downstream signaling molecules. As shown by dot blotting analysis, the effect of suramin on PA-SMCs stimulated with PDGF, FGF2 or EGF (10 ng/mL) was to reduce RTK phosphorylation levels. This inhibition was greatest for the PDGF receptors α and β, which are the predominant RTKs expressed in PA-SMCs, followed by FGF-R1 and EGF-R. The differences in the degree of inhibition may be explained by the expression levels of these receptors or by the specificity of binding to the receptor [25]. The mitogen-activated protein kinase (MAPK) cascade is the essential effector cascade required for most RTK functions. This cascade is composed of Raf, MEK, and extracellular signal-regulated kinase (ERK). Here, PDGF, FGF2, and EGF treatment of PA-SMCs resulted in rapid ERK1/2 activation, and this effect was completely blocked by suramin without changes in total ERK1/2 levels. These data raise the question of whether PA-SMCs in their native vascular environment might also respond to these stimuli and inhibitors. To investigate this, we cultured intact human pulmonary arteries to evaluate the effects of suramin treatment on vascular wall remodeling. The effects of suramin were compared with those of masitinib, which is an inhibitor of PDGF-R. Our results showed that wall thickness, PA-SMC proliferation and collagen deposition were significantly increased in pulmonary arteries incubated for ten days with 10% FCS. Treatment of the tissue explants with suramin was found to have a preventive effect on arterial vascular remodeling when vascular wall area, collagen deposition and α-smooth muscle actin staining were assessed, and this effect was not observed in masitinib-treated explants. Thus, the results obtained from our human pulmonary artery organ culture model confirm our cell culture observations and provide further evidence of the inhibitory effect of suramin on cell proliferation and consequently on vascular remodeling.

In this work, our ultimate purpose was to investigate the efficacy of suramin treatment for PH by using a PH model induced by monocrotaline administration in rats. In this model the pulmonary hypertension is severe and irreversible and is associated with prominent medial hypertrophy, inflammatory adventitial remodeling and, initially, pulmonary edema, endothelial apoptosis and growth factor upregulation [26]. We found that treatment of MCT-injected rats with suramin was followed by marked attenuation of PH development. This was indicated by a decrease in the levels of PAP, RV hypertrophy, and distal pulmonary artery muscularization compared with those of vehicle-treated rats when assayed at day 21. These data constitute strong evidence that suramin prevented the early upregulation of growth factors in response to MCT. Furthermore, we found that suramin treatment from day 21 to day 42 completely reversed distal vessel muscularization and medial wall hypertrophy in the pulmonary arteries, which suggests that increased growth factor expression may be necessary for both the progression and the maintenance of MCT-induced PH. 

Excessive PA-SMC proliferation is a hallmark of PH, and in this study we focused on the growth-promoting effects of PDGF, FGF2 and EGF in PA-SMCs and observed strong inhibition of SMC proliferation by suramin. However, we cannot ignore the fact that suramin acts on other cellular targets that act to enhance its inhibitory effect on RTKs. Previous studies [27,28] reported that suramin not only inhibits growth factor binding to SMCs by inducing conformational changes in the growth factors that decrease their ability to bind to the receptor but also displaces growth factors that are bound to the cell surface. Sjölnud et al. [29] showed that suramin induces upregulation of the cell surface expression of several receptors, including those for PDGF and FGF2, while inhibiting their intracellular internalization and degradation by reducing lysosomal enzyme activity. This suggests that proteolytic processing of either the growth factor or its receptor, or both, plays an important role in the generation of the mitogenic signal. In contrast, treatment of PA-SMCs with suramin did not affect the mRNA expression levels of DNA topoisomerase II and the ryanodine receptor (data not shown). Our results clearly suggest that pharmacological intervention targeting multiple growth factor signaling pathways would be a promising and practical approach for preventing PA-SMC proliferation and pulmonary vascular remodeling, both of which are considered at present to be central factors in all forms of human pulmonary hypertension.
